# The factors associated with the trend in incidence of Bacteraemia and associated mortality over 30 years

**DOI:** 10.1186/s12879-023-08018-0

**Published:** 2023-02-03

**Authors:** J. F. García-Rodríguez, A. Mariño-Callejo

**Affiliations:** 1 Infectious Diseases Unit, Department of Internal Medicine, University Hospital of Ferrol, Sergas. Ferrol, 15405 A Coruña, Spain; 2 C/ San Amaro 10-12, 6º Derecha, Ferrol, 15403 A Coruña, Spain

**Keywords:** Bacteraemias, Nosocomial, Community acquired, Epidemiology, Morbidity

## Abstract

**Background:**

Studies have reported increased incidence of BSI over the past decades and indicate that it is necessary to investigate the causes. The aim of this study was to determine the factors affecting trends in the incidence of bacteraemias and associated mortality.

**Methods:**

We conducted a retrospective cohort study assessing prospectively collected data of all clinically significant bacteraemias between 1991 and 2020 in a 450-bed hospital. We determined the evolution of bacteraemia-associated incidence, adjusted 30-day mortality and performed multivariable logistic regression to compare the evolution of variables associated with mortality between 5-year periods.

**Results:**

6777 episodes were included, 59.7% males, age 66.5 ± 18.2, 39.4% ≥ 75 years. The incidence total increased: 43.8/100,000/year in 1991–1995 to 205 in 2016–2020; community-acquired bacteraemia (24.9 to 139) and hospital-acquired (0.36/1000 inpatients-days to 1.09). Bacteraemia with source in vascular catheter, urinary and biliary tract increased. The 30-day mortality rate of patients was 1179/6777 (17.4%) in the whole series and population-adjusted mortality incidence increased from 11.4/100,000 in 1991–1996 to 28.4 in 2016–2020 (RR 2.49, 95% CI 2.01–3.08). Mortality was higher in men (18.2% vs 16.3%) and those over 74 years (22.2% vs 14.3%). Appropriate empirical antimicrobial treatment improved (66.5% to 73.1%), 30-day mortality of patients decreased from 26.1 to 13.9%. When comparing the evolution of the factors associated with mortality between 1991 and 1996 vs 2016–2020, the frequency of some variables associated with higher mortality increased: male sex (OR 1.38, 95% CI 1.10–1,74), age (OR 1.02, 1.01–10.3), immunosuppressive treatment (OR 3.1, 2.09–4.6), polymicrobial bacteraemia (OR 1.76, 1.12–2.79), and others decreased: severe sepsis/septic shock (OR 0.70, 0.52–0.93), spontaneous bacterial peritonitis in cirrhosis (OR 0.06, 0.02–0.23), endocarditis (OR 0.54, 0.35–0.83); on the other hand, the frequency of factors associated with lower mortality increased: urinary (OR 1.67, 95% CI 1.23–2.27) and bile tract source (OR 1.59, 1.04–2.43), and adequate empirical treatment (OR 1.42, 95% CI 1.10–1.83).

**Conclusions:**

The incidence of bacteraemia increased due to more elderly, co-morbid patients undergoing procedures and more device related bacteraemia. The percentage of mortality decreased because adequate empirical treatment improved, decreased spontaneous bacterial peritonitis in cirrhosis and endocarditis, and increased bacteraemia of urinary and biliary tract source.

## Introduction

Bloodstream infections (BSI) are a significant cause of morbidity and mortality worldwide [1]. An estimated near 2 million episodes and 250,000 deaths from BSI occur annually in North America and Europe combined. It is conceivable that annual deaths from BSI may be comparable those caused by the three primary infectious diseases killers in the world: human immunodeficiency virus, tuberculosis, and malaria [2]. Over the past decades, studies in Denmark and Finland, using data from the national registries, have reported increased incidence of BSI, probably associated with an increase in the number of blood cultures performed, successive improvements of blood culture techniques and an increase in the population at risk [3–5].

Diagnosis of BSI requires the detection, growth and isolation of the micro-organism in blood cultures. Sampling is not always possible as early as desired, a positive blood culture result can range depending on the focus of infection from 1 to 50%, and microbiological results can take hours to a few days [6, 7]. For these reasons, empirical treatment protocols are necessary to improve clinical results and reduce mortality [8]. These protocols should be based on the epidemiology of the infection, the most likely aetiological agent, source of infection, place of acquisition, patient comorbidities, baseline situation, and the frequency of bacterial resistance to antibiotics.

Epidemiological studies have revealed the most common pathogens isolated in BSI episodes according to place of acquisition or focus of infection, they are however limited to type of infection, microorganism, or over short periods of time [9, 10]. Clinical practice of performing blood cultures may differ in both institutions and geographical areas and change over time [11]. There are very few long-term studies depicting the evolution of the epidemiology and clinical features of bacteremic infection. It is necessary to have knowledge of local data, documenting the burden of disease caused by BSI and its epidemiology, as well as changes over time to guide therapy, plan healthcare delivery and research resources.

The aim of this study was to investigate the factors affecting trends in the incidence of bacteraemia and associated mortality.

## Methods

### Design, study site and periods

We conducted a retrospective cohort study assessing prospectively collected data, with a protocol specifically designed in 1990 for the follow-up of all clinically significant bacteraemias, between 1991 and 2020 in a 450-bed hospital located in the northwest of Spain. Generally the north west has an aged population, cold winter and damp conditions. BSI were detected by daily review of blood culture results. The hospital provides healthcare for an area with 200,000 inhabitants and has an ICU with 10 beds. Patient samples were taken as part of standard care. Blood cultures were obtained, processed and interpreted in accordance with standard recommendations [12].

The patients were followed up 30 days after the episode or death by reviewing patients´ clinical documentation. A Computerised database for data capturing was created. Participants were unidentifiable, and only health professionals had access to the information, maintaining strict confidentiality. The study was approved by the ethics committees of our hospital, owing to the observational design of the study, the need to obtain informed consent was waived. The Strengthening the Reporting of Observational Studies in Epidemiology (STROBE) reporting guideline was followed.

### Variables and definitions

Data was collected for all episodes by a single Infectious Diseases specialist according to a specifically designed protocol and included the following information: admitting ward, demographics, neutropenia (defined as an absolute neutrophil count of < 1000 cells/μL) site of infection, type of acquisition [community-acquired (CA) or hospital-acquired (HA)], severity of underlying diseases [13], exposure to invasive procedures and devices (including vascular or urinary catheter), antimicrobial use in preceding 7 days before BSI; source of infection, aetiology, clinical situation of patient presentation with severe sepsis/septic shock according to classic [14] and Sepsis-3 [15] criteria, empirical antimicrobial treatment, and 30-day mortality. Episodes were classified as HA if they occurred > 48 h after admission, or < 48 h if the patient had been hospitalised in the previous two weeks, otherwise, they were classified as CA.

The BSI source was determined considering clinical and laboratory data using classic US Centres for Disease Control and Prevention (CDC) criteria for secondary BSI [16]. BSIs were considered clinically significant if accompanied by signs or symptoms of infection, and contamination was excluded. For potential contaminants, such as coagulase-negative staphylococci (CoNS) or diphtheroids, only episodes in which the organism was isolated from at least two blood draws were considered. Patients with recurrent isolation of the same microorganisms were considered as a single episode of BSI unless the sample was obtained one month after the last positive blood culture.

We defined polymicrobial bacteraemia as an episode with detection of more than one clinically significant blood culture isolate within 48 h; ESBL-*E. coli* and ESBL-*K. pneumoniae* as resistant or intermediately sensitive to third-generation cephalosporin; CPE as *E. coli*, *K. pneumoniae*, and *Enterobacter spp*. resistant or intermediately sensitive to carbapenems. We defined empirical antimicrobial treatment as the antibiotic treatment given at the 1st notification of a positive blood culture; it was recorded as appropriate if all the blood isolates were sensitive to one or more of the antibiotics given. There were no definition changes during the study period.

Total BSI incidence and CA BSI cases/100,000 inhabitants-year (data from population census), and HA infection/1000 inpatient-days (obtained from administrative data) were calculated. From 1991, the number of vascular and urinary catheter use was determined by prevalence studies.

### Statistical analysis

A descriptive and comparative study of the variables was performed. Quantitative variables are reported as mean ± SD, and categorical as frequencies (%). Categorical variables were compared using crosstabs for 2 or more groups with Chi-square test or Fisher exact test, and the relative risk calculated with 95% confidence interval (CI). Student t-test or Mann–Whitney U for continuous variables, as appropriate. To compare continuous variables for 3 or more groups we use one-way ANOVA, and then conduct post-hoc analysis with Bon-Ferrini's method. Patients’ characteristics were compared according to the type of acquisition. The 30-year duration was divided into 5-year periods to compare the evolution of the variables analyzed over time. The patients were divided by age group to compare the distribution of sources and aetiology of BSIs.

HA rates per 1,000 inpatient-days and CA and total rates per 100,000 inhabitants-year with a 95% CI were calculated as Poisson event rates. Logistic regression was performed to identify variables associated with 30-day death in BSIs using stepwise analysis. We included all variables associated with the dependent variable on bi-variable analysis (p < 0.1), and pre-defined variables that made clinical sense. Associations between the variables were expressed as odds ratios (ORs) and 95% confidence intervals (CIs). Statistical analysis was performed using IBM SPSS Statistics v.23.0. Two-sided p-values < 0.05 was considered statistically significant.

## Results

### Incidence, underlying conditions and predisposing features.

During the study period, a total of 6777 episodes were included. The incidence of bacteraemia increased to 205.1/100,000 population in 2016–2020 vs 43.8 in 1991–1995 (RR 4.68, 95% CI 4.23–5.17). Out of the total, 4043 (59.7%) occurred in males, mean age 66.5 ± 18.2 (range 0–99 years), 39.4% ≥ 75 years old. The clinical specialties most commonly diagnosing BSI were General Medicine (44.1%), General Surgery (16.1%) and ICU (9%). The number of blood cultures performed per 1,000 inpatients-days increased from 17.1 in 1998 to 38.2 in 2020 (RR 2.23, 95% CI 2.13–2.33), with the percentage of positive blood cultures ranging between 8.1 and 10.5%.

The evolution of incidence and overall patient characteristics are shown in Table 1. BSI sources such as pneumonia (12.2% vs 9.9%), endocarditis (5.5% vs 3.5%) and urinary catheterisation (9.3% vs 2.8%) was more common in males, urinary aetiology without catheter was more common in females (27.9% vs 23.4%, p < 0.001). Bacteraemia of unknown source decreased throughout the study period. Increased incidence was associated with increasing age, male, comorbidities (diabetes mellitus, cancer), McCabe fatal-rapidly fatal, immunosuppressive treatment, and instrumentation increased over the study period. Use of vascular catheters (27.4% in 1991–1995 vs 75.1% in 2016–2020) and urinary catheters (8.4% vs 14.5%, p < 0.001) increased. The bacteraemia that occurring in males after perianal prostate biopsy accounted for 3.2% of HA bacteraemias (*E. coli-* associated).

**Table 1:** Patient characteristics, incidence, acquisition, source of infection, aetiology, clinical features and mortality

Characteristics	Years						
	1991–1995	1996–2000	2001–2005	2006–2010	2011–2015	2016–2020	p
No episodes	476	676	790	1175	1705	1955	
Incidence per-100,000 population	43.8	63.5	76.1	77	170.8	205.1	< 0.001
Male nº (%)	264 (55.5)	404 (59.8)	462 (58.5)	727 (61.9)	1010 (59.2)	1176 (60.2)	0.247
Age, mean ± SD	62.1 ± 19.6	57.1 ± 23.9	63.5 ± 18.9	65.5 ± 18.2	68.7 ± 16.3	70.6 ± 15	< 0.05
McCabe fatal-rapidly fatal	185 (38.9)	335 (49.6)	404 (51.1)	545 (46.4)	888 (52.1)	1209 (61.8)	< 0.001
Urinary catheter	81 (17)	87 (12.9)	111 (14.1)	223 (19)	286 (16.8)	296 (15.1)	0.004
Vascular catheter	176 (37)	228 (33.4)	290 (36.7)	404 (34.4)	443 (26)	570 (29.1)	< 0.001
Parenteral nutrition	59 (12.4)	85 (12.6)	107 (13.5)	187 (15.9)	210 (12.3)	275 (14.1)	0.098
Mechanical ventilation	19 (4)	8 (1.2)	12 (1.5)	32 (2.7)	25 (1.5)	50 (2.6)	0.015
Previous antimicrobial therapy	119 (25)	176 (26)	232 (29.4)	332 (28.3)	424 (24.9)	492 (25.2)	0.085
Neutropenia	21 (4.5)	41 (6.1)	49 (6.2)	53 (4.5)	67 (3.9)	68 (3.5)	0.008
Injecting drug abuser	38 (8)	37 (5.5)	23 (2.9)	23 (2)	8 (0.5)	18 (0.9)	< 0.001
HIV	31 (6.5)	43 (6.4)	33 (4.2)	20 (1.7)	22 (1.3)	23 (1.2)	< 0.001
Diabetes mellitus	128 (26.9)	124 (18.3)	193 (24.4)	291 (24.8)	448 (26.3)	562 (28.7)	< 0.001
Cancer	89 (18.7)	183 (27.1)	225 (28.5)	347 (29.6)	482 (28.3)	579 (29.6)	< 0.001
Inmunosuppressive treatment	42 (8.8)	99 (14.6)	154 (19.5)	239 (20.3)	305 (17.9)	400 (20.5)	< 0.001
Surgery	80 (16.8)	118 (17.5)	143 (18.1)	222 (18.9)	240 (14.1)	240 (12.3)	< 0.001
Source							
Unknown	61 (12.8)	113 (16.7)	74 (9.4)	99 (8.4)	131 (7.7)	110 (5.6)	< 0.001
Urinary tract	97 (20.4)	114 (16.9)	178 (22.5)	270 (23)	511 (30)	624 (31.9)	< 0.001
Vascular catheter	78 (16.4)	115 (17)	150 (19)	235 (20)	240 (14.1)	339 (17.3)	0.001
Respiratory tract	56 (11.8)	104 (15.4)	113 (14.3)	149 (12.7)	164 (9.6)	193 (9.9)	< 0.001
Biliary tract	37 (7.8)	50 (7.4)	72 (9.1)	146 (12.4)	274 (16.1)	289 (14.8)	< 0.001
Intra-abdominal	22 (4.6)	24 (3.6)	47 (5.9)	47 (4)	76 (4.5)	64 (3.3)	0.041
Endocarditis	44 (9.2)	46 (6.8)	39 (4.9)	61 (5.2)	60 (3.5)	72 (3.7)	< 0.001
Skin and soft tissues	16 (3.4)	39 (5.8)	27 (3.4)	49 (4.2)	71 (4.2)	79 (4)	0.275
Osteoarticular	7 (1.5)	7 (1)	14 (1.8)	14 (1.2)	24 (1.4)	24 (1.2)	0.835
Other	136 (28.6)	179 (26.5)	226 (28.6)	340 (28.9)	394 (23.1)	500 (25.6)	0.004
Aetiology							
Polymicrobial bacteraemia	29 (6.1)	54 (8)	76 (9.6)	132 (11.2)	161 (9.4)	217 (11.1)	0.009
Monomicrobial bacteraemia	447 (93.9)	622 (92)	714 (90.4)	1043 (88.8)	1544 (90.6)	1738 (88.9)	0.005
Gram-negative bacteria	240 (50.4)	335 (49.6)	384 (48.6)	598 (50.9)	982 (57.6)	1114 (57)	< 0.001
*E. coli*	123 (25.8)	190 (28.1)	250 (31.6)	403 (34.3)	699 (41)	736 (37.6)	< 0.001
*Klebsiella* spp.	14 (2.9)	22 (3.3)	37 (4.7)	50 (4.3)	94 (5.5)	115 (5.9)	0.013
*Proteus* spp.	21 (4.4)	12 (1.8)	19 (2.4)	25 (2.1)	37 (2.2)	53 (2.7)	0.06
*Pseudomonas* spp.	13 (2.7)	11 (1.6)	17 (2.2)	34 (2.9)	39 (2.3)	52 (2.7)	0.566
Gram-positive bacteria	201 (42.2)	291 (43)	344 (43.5)	501 (42.6)	615 (36.1)	744 (38.1)	< 0.001
*S. aureus*	66 (13.9)	77 (11.4)	88 (11.1)	115 (9.8)	148 (8.7)	148 (7.6)	< 0.001
*Coagulase-negative staphylococci*	57 (12)	87 (12.9)	103 (13)	180 (15.3)	190 (11.1)	261 (13.4)	0.043
*Strep. pneumoniae*	23 (4.8)	59 (8.7)	73 (9.2)	88 (7.5)	80 (4.7)	94 (4.8)	< 0.001
*Enterococcus* spp.	16 (3.4)	21 (3.1)	20 (2.5)	31 (2.6)	58 (3.4)	82 (4.2)	0.163
Anaerobes	23 (4.8)	31 (4.6)	40 (5.1)	37 (3.1)	46 (2.7)	52 (2.7)	0.002
*Candida* spp.	9 (1.9)	6 (0.9)	15 (1.9)	22 (1.9)	39 (2.3)	30 (1.5)	0.269
Resistant bacteria							
Methicillin-resistant S. aureus*	1/66 (1.5)	2/77 (2.6)	12 /88 (13.6)	13/115 (11.3)	25/148 (16.9)	14/148 (9.5)	0.002
ESBL-producing *Enterobacteriales*^*#*^	2/209 (1)	4/259 (1.5)	14/343 (4.1)	49/525 (9.3)	107/899 (11.9)	120/998 (12)	< 0.001
Severe sepsis/septic shock	100 (21)	116 (17.2)	140 (17.7)	220 (18.7	380 (22.3)	371 (19)	0.017
Appropriate EAT	302 (63.4)	452 (66.9)	526 (66.6)	793 (67.5)	1276 (74.8)	1398 (71.5)	< 0.001
30-day mortality	124 (26.1)	141 (20.9)	179 (22.7)	212 (18)	252 (14.8)	271 (13.9)	< 0.001

Date are presented as n (%) unless otherwise indicated

*EAT* empirical antimicrobial treatment

*Proportion among *S. aureus*

^#^Proportion among Enterobacteriales

**Fig. 1:**
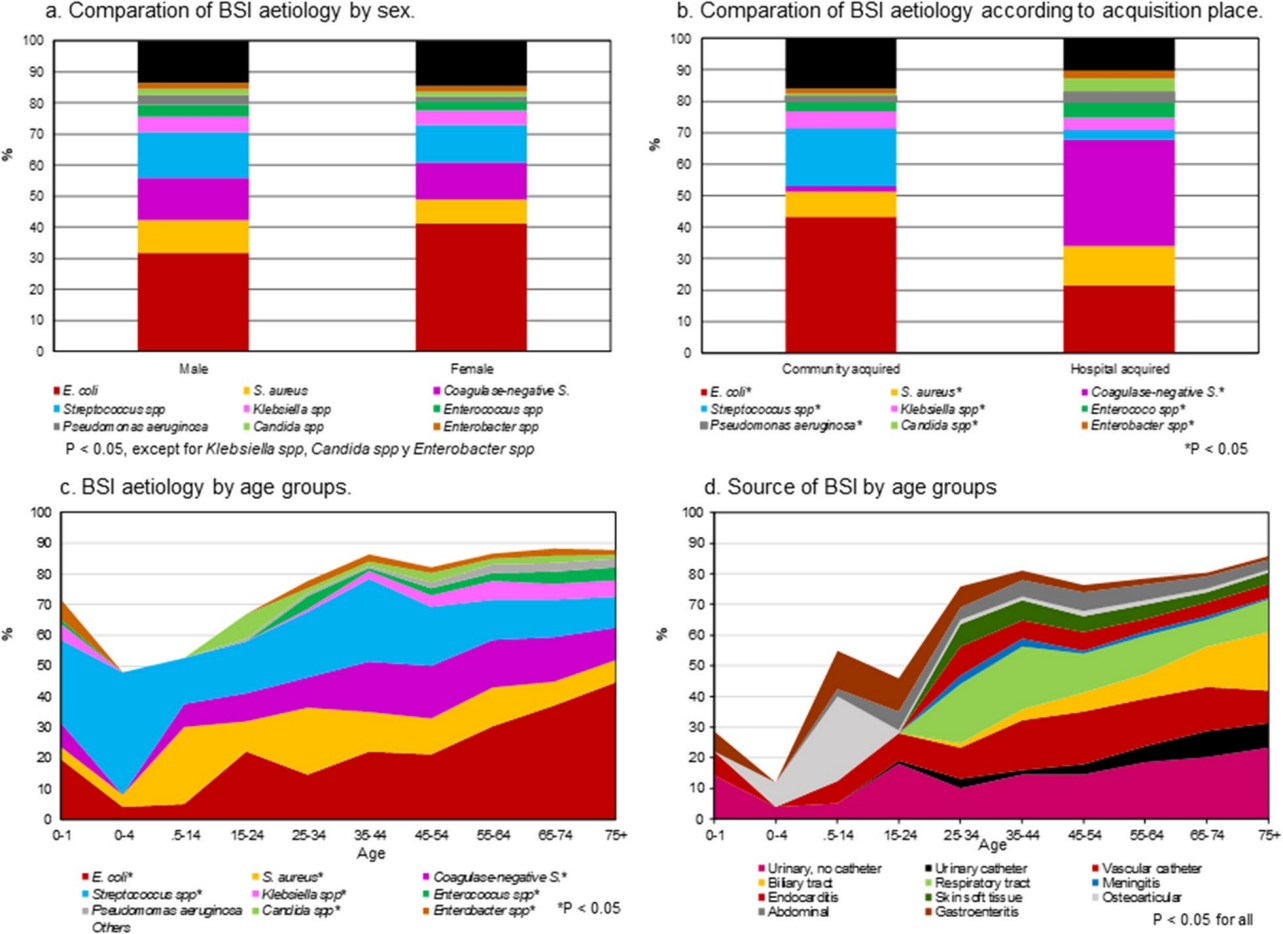
The isolated microorganisms according to sex, acquisition place, age. Source of BSI by age groups

The isolated microorganisms differed in frequency according to patient age, sex, and place of acquisition of infection. The frequency of bacteraemia increased with patient age and the sources differed between age groups (Fig. 1). An overall increase in Gram-negative microorganisms and polymicrobial bacteraemia was seen in CA-BSI; Gram-positive microorganisms decreased in CA-BSI and increased in HA-BSI. The isolation of *E. coli*, *Klebsiella spp* and CoNS increased, the isolation of *Salmonella typhi* decreased (1.6 per 100,000 population in 1991–1995 to 0 in 2006–2020), and *S. aureus* also decreased in the last 15 years, p < 0.05. The isolation of ESBL-producing Enterobacters increased progressively, and the isolation of MRSA decreased in the last 5 years. Carbapenemase-producing microorganisms were not identified in BSI. Polymicrobial bacteraemia occurred in 669 (10%) patients and increased throughout the study period (RR in 2016–2020 vs 1991–1995: 7.4, 95% CI 5–10.9).

### Comparations between CA and HA bacteraemia

The type of acquisition was CA 4,430 (65.4%; 95% CI 64–66.8) and HA 2.347 (34.6%; 95% CI 31.3–37.9). Table 2 shows the incidence and patient characteristics according to the type of acquisition. The incidence of CA bacteraemia increased (RR 2016–2020 vs 1991–1995: 5.6, 95% CI 4.9–6.4). There were more bacteraemia originating in the urinary and biliary tract. Urinary tract (28.6% vs 24.7%) or gastrointestinal (2.7% vs 1.3%) sources were more frequent in June-December, whereas pneumonia as a source was more common in December-March (16.1% vs 9.4%), p < 0.001.

**Table 2:** Comparison between CA and HA in patient characteristics, acquisition, incidence, source of infection, aetiology, clinical features, and mortality

Characteristic	Years						
	1991–1995	1996–2000	2001–2005	2006–2010	2011–2015	2016–2020	p
Community acquired 4430 (65.4)	270	414	449	720	1252	1325	
Incidence per 100,000 population	24.9	38.9	43.2	70.2	125.4	139.0	< 0.05
Male no (%)	138 (51.1)	246 (59.4)	258 (57.5)	421 (58.5)	722 (57.7)	746 (56.3)	0.311
Age, mean ± SD	59.9 ± 21.6	54.1 ± 26.1	61.9 ± 21.1	65.8 ± 19.6	69.2 ± 16.7	71.2 ± 15.5	< 0.05
McCabe fatal-rapidly fatal	90 (33.3)	176 (42.5)	206 (45.9)	313 (43.5)	619 (49.4)	787 (59.4)	< 0.001
Urinary catheter	15 (5.6)	31 (7.5)	22 (4.9)	55 (7.6)	95 (7.6)	91 (6.9)	0.389
Previous antimicrobial therapy	35 (13)	49 (11.8)	62 (13.8)	75 (10.4)	141 (11.3)	117 (8.8)	0.039
Neutropenia	11 (4.1)	34 (8.3)	32 (7.1)	42 (5.9)	52 (4.2)	49 (3.7)	0.001
Injecting drug abuser	34 (12.6)	29 (7)	20 (4.5)	17 (2.4)	7 (0.6)	15 (1.1)	< 0.001
HIV	27 (10)	32 (7.7)	24 (5.3)	14 (1.9)	18 (1.4)	19 (1.4)	< 0.001
Diabetes mellitus	72 (26.7)	76 (18.4)	100 (22.3)	185 (25.7)	338 (27)	397 (30)	< 0.001
Cancer	34 (12.6)	88 (21.3)	91 (20.3)	169 (23.5)	288 (23)	316 (23.8)	0.002
Inmunossuppressive treatment	13 (4.8)	56 (13.5)	73 (16.3)	130 (18.1)	199 (15.9)	227 (17.1)	< 0.001
Source more frequenty							
Unknown	27 (10)	45 (10.9)	46 (10.2)	72 (10)	104 (8.3)	83 (6.3)	< 0.001
Urinary tract	67 (24.8)	85 (20.5)	118 (26.3)	200 (27.8)	434 (34.7)	511 (38.6)	< 0.001
Respiratory tract	42 (15.6)	80 (19.3)	88 (19.6)	121 (16.8)	142 (11.3)	156 (11.8)	< 0.001
Biliary tract	28 (10.4)	29 (7)	47 (10.5)	121 (16.8)	242 (19.3)	258 (19.5)	< 0.001
Endocarditis	38 (14.1)	37 (8.9)	38 (8.5)	48 (6.7)	52 (4.2)	60 (4.5)	< 0.001
Aetiology, most frequent							
Polymicrobial bacteraemia	11 (4.1)	28 (7.1)	45 (10)	76 (10.6)	131 (10.5)	160 (12.1)	0.001
Monomicrobial bacteraemia	259 (95.9)	386 (93.2)	404 (90)	644 (89.4)	1121 (89.5)	1165 (87.9)	0.001
Gram-negative bacteria	157 (58.1)	234 (56.5)	242 (53.9)	442 (61.4)	822 (65.7)	881 (66.5)	< 0.001
*E. coli*	88 (32.6)	125 (30.2)	162 (36.1)	318 (44.2)	593 (47.4)	613 (46.3)	< 0.001
*Klebsiella* spp.	6 (2.2)	12 (2.9)	23 (5.1)	33 (4.6)	78 (6.2)	87 (6.6)	0.005
*Proteus* spp.	12 (4.4)	9 (2.2)	15 (3.3)	16 (2.2)	31 (2.5)	42 (3.2)	0.332
Gram-positive bacteria	102 (37.8)	152 (36.7)	178 (39.6)	238 (33.1)	368 (29.4)	380 (28.7)	< 0.001
*S. aureus*	38 (14.1)	39 (9.4%)	43 (9.6%)	53 (7.4%)	95 (7.6%)	78 (5.9%)	< 0.001
*Strep. pneumoniae*	23 (8.5)	54 (13)	68 (15.1)	82 (11.4)	77 (6.2)	90 (6.8)	< 0.001
*Enterococcus* spp.	4 (1.5)	11 (2.7)	8 (1.8)	12 (1.7)	35 (2.8)	48 (3.6)	0.066
Severe sepsis/septic shock	56 (20.7)	80 (19.3)	92 (20.5)	148 (20.6)	285 (22.8)	239 (18)	0.101
Appropriate EAT	200 (75.5)	315 (78.6)	349 (78.4)	547 (76.9)	1021 (82.3)	1032 (79.1)	0.037
30-day mortality	59 (21.9)	79 (19.1)	95 (21.2)	118 (16.4)	166 (13.3)	155 (11.7)	< 0.001
Hospital acquired 2347 (34.6)	206	262	341	455	453	630	
Incidence per 1000 inpatients-days	0.363	0.466	0.612	0.753	0.762	1.098	< 0.05
Male nº (%)	126 (61.2)	158 (60.3)	204 (59.8)	306 (67.3)	288 (63.6)	430 (68.3)	0.028
Age, mean ± SD	64.9 ± 16.1	61.7 ± 18.9	65.6 ± 15.5	65 ± 15.9	67.5 ± 14.8	69.4 ± 14	< 0.05
McCabe fatal-rapidly fatal	95 (46.1)	159 (60.7)	198 (58.1)	232 (51)	269 (59.4)	422 (67)	< 0.001
Urinary catheter	66 (32)	56 (21.4)	89 (26.1)	168 (35.9)	191 (42.2)	205 (32.5)	< 0.001
Vascular catheter	140 (68)	183 (69.8)	247 (72.4)	352 (77.4)	359 (79.2)	516 (81.9)	< 0.001
Parenteral nutrition	59 (28.6)	84 (32.1)	105 (30.8)	182 (40)	193 (42.6)	271 (43)	< 0.001
Mechanical ventilation	17 (8.3)	7 (2.7)	12 (3.5)	32 (7)	22 (4.9)	48 (7.6)	0.007
Previous antimicrobial therapy	84 (40.8)	127 (48.5)	170 (49.9)	257 (56.5)	283 (62.5)	375 (59.5)	< 0.001
Neutropenia	10 (5.2)	7 (2.7)	17 (5)	11 (2.4)	15 (3.3)	19 (3)	0.258
Injecting drug user	4 (1.9)	8 (3.1)	3 (0.9)	6 (1.3)	1 (0.2)	3 (0.5)	0.005
HIV	4 (1.9)	11 (4.2)	9 (2.6)	6 (1.3)	4 (0.9)	4 (0.6)	0.002
Diabetes mellitus	56 (27.2)	48 (18.3)	93 (27.3)	106 (23.3)	110 (24.3)	165 (26.2)	0.112
Cancer	55 (26.7)	95 (36.3)	134 (39.3)	178 (39.2)	194 (42.8)	263 (41.7)	0.002
Inmunossuppressive therapy	29 (14.1)	43 (16.4)	81 (23.8)	109 (24)	106 (23.4)	173 (27.5)	< 0.001
Surgery	71 (34.5)	113 (43.1)	134 (39.3)	213 (46.8)	213 (47)	232 (36.8)	0.001
Source							
Unknown	34 (16.5)	28 (10.7)	28 (8.2)	27 (5.9)	27 (6)	27 (4.3)	< 0.001
Urinary tract	30 (14.6)	29 (11.1)	60 (17.6)	70 (15.4)	77 (17)	113 (17.9)	0.166
Vascular catheter	78 (37.9)	112 (42.7)	146 (42.8)	229 (50.3)	216 (47.7)	318 (50.5)	0.006
Respiratory tract	14 (6.8)	24 (9.2)	25 (7.3)	28 (6.2)	22 (4.8)	37 (5.9)	0.304
Biliary tract	9 (4.4)	21 (8)	25 (7.3)	25 (5.5)	32 (7.1)	31 (4.9)	0.285
Aetiology, most frequent							
Polymicrobial bacteraemia	18 (8.7)	26 (9.9)	31 (9.1)	56 (12.3)	30 (6.6)	57 (9.1)	0.104
Monomicrobial bacteraemia	188 (91.3)	236 (90.1)	310 (90.9)	399 (87.7)	423 (93.4)	573 (90.9)	0.104
Gram-negative bacteria	83 (40.3)	101 (38.5)	142 (41.6)	156 (34.3)	160 (35.3)	233 (37)	0.281
*E. coli*	35 (17)	65 (24.8)	88 (25.8)	85 (18.7)	106 (23.4)	123 (19.5)	0.028
*Klebsiella* spp.	8 (3.9)	10 (3.8)	14 (4.1)	17 (3.7)	16 (3.5)	28 (4.4)	0.983
*Pseud. aeruginosa*	8 (3.9)	6 (2.3)	10 (2.9)	20 (4.4)	16 (3.5)	23 (3.7)	0.755
Gram-positive bacteria	99 (48.1)	139 (53.1)	166 (48.7)	263 (57.8)	247 (54.5)	364 (57.5)	0.023
*S. aureus*	28 (13.6)	38 (14.5)	45 (13.2)	62 (13.6)	53 (11.7)	70 (11.1)	0.671
Coagulase-negative staphylococci	51 (24.8)	77 (29.4)	99 (29)	167 (36.7)	162 (35.8)	237 (37.6)	0.001
*Enterococcus* spp.	12 (5.8)	10 (3.8)	12 (3.5)	19 (4.2)	23 (5.1)	34 (5.4)	0.667
Severe sepsis/septic shock	44 (21.4)	36 (13.7)	48 (14.1)	72 (15.8)	95 (21)	132 (21)	0.007
Appropriate EAT	102 (54)	137 (54.4)	177 (53)	246 (55.5)	255 (58.2)	366 (60.1)	0.259
30-day mortality	65 (31.6)	62 (23.7)	84 (24.6)	94 (20.7)	86 (19)	116 (18.4)	0.001

Date are presented as n (%) unless otherwise indicated. EAT: empirical antibiotic treatment

*E. coli*, *S. aureus* and *Streptococcus pneumoniae* are the most common microorganisms. The isolated microorganism varies according to bacteraemia source. *E. coli* is more commonly associated with urinary (55%) and biliary tract (25.3%); *Proteus mirabilis* in urinary tract (74.4%); *Klebsiella spp* in biliary (40.6%) and urinary tract (35.1%); *S. aureus* in endocarditis (21.4%), skin and soft tissue (15.1%) and pneumonia (14.2%); *Enterococcus *spp. in urinary tract (28.8%) and endocarditis (28.8%); *S. pneumoniae* in pneumonia (83%) or meningitis source (9.4%). *E coli* is more frequent in June-October months (38.5 vs 32.9, p < 0.01) and *S. pneumoniae* in December–March (50.8% vs 30.2%, p < 0,001). The incidence of pneumococcal bacteraemia increased and resistance to penicillin decreased since the first vaccination campaign introduced in 2000 (resistance to penicillin 27.1% in 1996–2000 vs 3.2% in 2016–2020).

The incidence of HA bacteraemia increased (RR 2016–2020 vs 1991–1995: 3, 95% CI 2.6–3.5), especially that of vascular catheter (RR 2016–2020 vs 1991–1995: 4, 95% CI 3.1–5.2) and urinary tract source (RR 2016–2020 vs 1991–1995: 3.7, 95% CI 2.5–5.6).

CoNS, *E. coli*, *S. aureus*, *Candida spp*, *Pseudomonas aeruginosa* and *Enterococcus spp* are the most frequent microorganisms. The inpatient length-of-stay before presentation of bacteraemia ranged from 11 days for *E. coli* and S*. aureus* to 18 for *Pseudomonas aeruginosa* and *Klebsiella spp,* and 22 for *Candida *spp. The isolated microorganism varied according to bacteraemia source, CoNS (93.8%), *S. aureus* (57.8%) and *Candida *spp. (66%) in vascular catheter; *E. coli* (46.2%), *Klebsiella* spp. (22.6%) and *Enterococcus* spp. (26.4%) in urinary tract, and *Pseudomonas aeruginosa* (32.5%) in pneumonia.

### Severity at presentation and outcome

Overall, 1327/6777 (19.6%, 95% CI 18.7–20.5) of BSI episodes presented as severe sepsis/septic shock, 900/4430 (20.3%, 95%CI 17.4–23.2) for CA-BSI and 427/2347 (18.2%, 95%CI 16.5–19.9) for HA-BSI. The proportion of patients with severe sepsis/septic shock did not differ between patients with CA or HA bacteraemia and increased throughout the study period in HA bacteraemia.

**Fig. 2:**
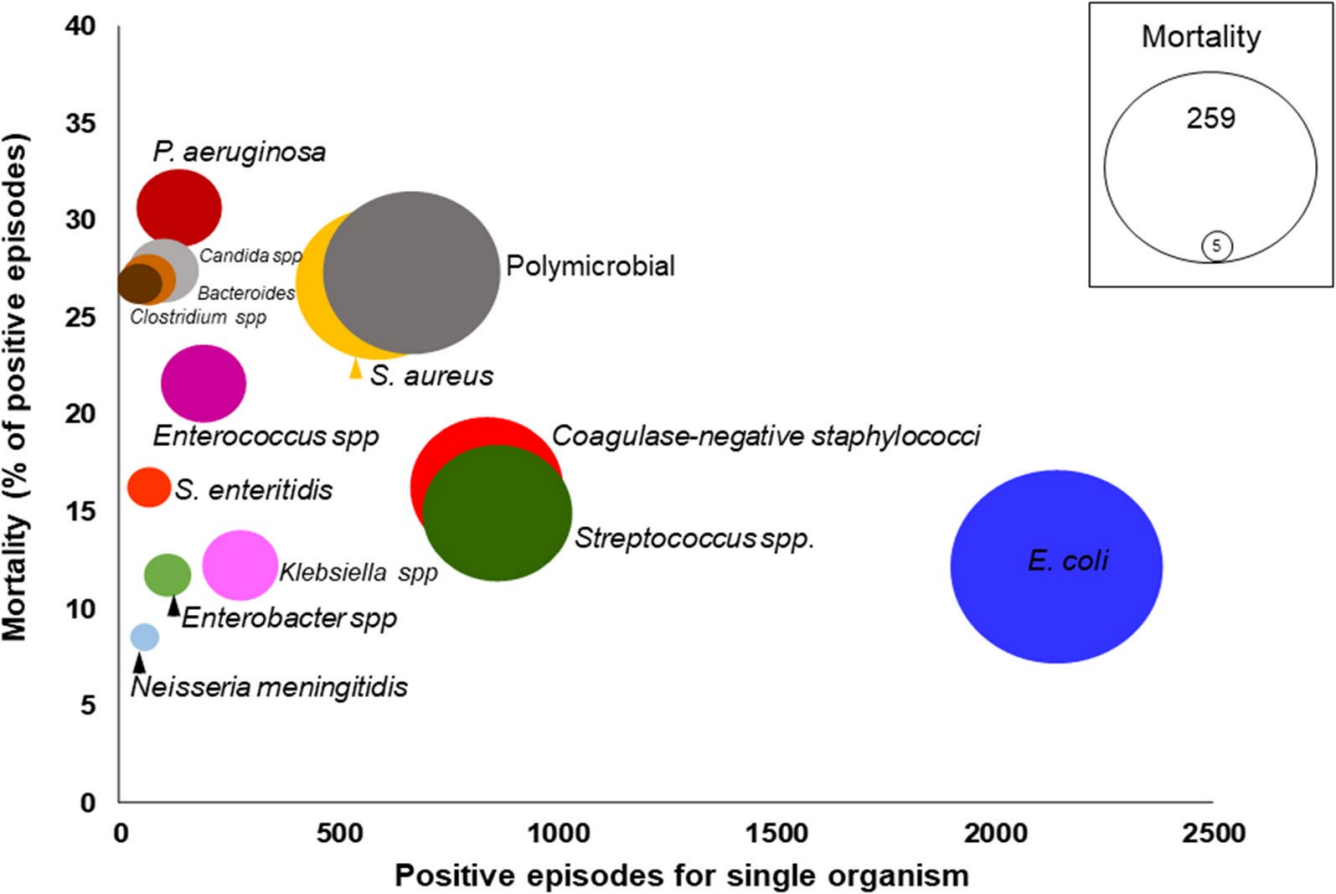
Incidence and lethality of bloodstream infection (BSI) organisms

The 30-day mortality rate of patients was 1179/6777 (17.4%) in the whole series and population-adjusted mortality incidence increased from 11.4/100,000 population in 1991–1996 to 28.4 in 2016–2020 (RR 2.49, 95% CI 2.01–3.08). Mortality was higher in men (18.2% vs 16.3%; OR 1.14, 95% CI 1.01–1.30) and in patients over 74 years-old (22.2% vs 14.5%, OR 1.7, 95% CI 1.5–1.9). Mortality was higher in HA-BSI 507/2347 (21.6%, 95% CI 19.9–23.3) than CA-BSI (672/4430 (15.2%, 95% CI 14.2–16.2), p < 0.001, and was different depending on the causative microorganism (Fig. 2). The factors associated with mortality in the logistic regression analysis are shown in Table 3. Appropriate empirical antibiotic treatment improved throughout the study period and percentage of mortality decreased.

**Table 3:** Multivariable analysis of factors associated with 30-day mortality

Variables	OR (95% CI)	p
Male	1.07 (0.92–1.24)	0.390
Age	1.025 (1.02–1.03)	< 0.001
Severe sepsis/septic shock	4.28 (3.64–5.03)	< 0.001
Neutropenia	2.09 (1.56–2.80)	< 0.001
McCabe fatal-rapidly fatal	3.57 (2.98–4.28)	< 0.001
Source		
Respiratory tract	1.99 (1.60–2.50)	< 0.001
Intra-abdominal	2.37 (1.71–3.27)	< 0.001
Spontaneous bacterial peritonitis in cirrhotic	3.87 (2.14–7.01)	0.001
Meningitis	3.81 (2.14–6.77)	< 0.001
Endocarditis	2.50 (1.84–3.40)	< 0.001
Urinary tract	0.49 (0.40–0.61)	< 0.001
Biliary tract	0.53 (0.41–0.70)	< 0.001
Aetiology		
*S. aureus*	2.15 (1.71–2.70)	< 0.001
*Enterococcus* spp.	1.62 (1.13–2.33)	0.009
*Candida* spp.	2.19 (1.39–3.44)	0.001
Polymicrobial bacteraemia	1.61 (1.29–1.99)	< 0.001
Immunosuppressive treatment	1.28 (1.07–1.53)	0.008
Appropriate empirical antimicrobial treatment	0.76 (0.65–0.90)	0.002

Because co-morbidities can differ by acquisition place, we performed multivariate analyses to understand which demographic and underlying conditions were most prevalent in the different acquisition types. Compared with CA-BSI, HA patients were of similar age (66.4 ± 15.7 vs 66.5 ± 19.4); HA episodes were more frequent in males (OR 1.25, 95% CI 1.05–1.48), had higher rates vascular catheterisation (OR 31.6, 95% CI 26.5–37.7), urinary catheterisation (OR 1.77, 95% CI 1.38–2.26), surgery (OR 22.5, 95% CI 16.5–30.7), immunosuppressive treatment (OR 1.31, 95% CI 1.05–2.61) and exposure to antibiotics (OR 3.34, 95% CI 2.75–4.05), and less neutropenia (OR 0.56, 95% CI 0.38–0.84).

We also performed multivariate analyses using logistic regression to compare the evolution of the variables associated with mortality over five periods of time (1991–1995 reference; Table 4). Increased patient age, immunosuppressive treatment, polymicrobial bacteraemia and decreased neutropenia, endocarditis, spontaneous bacterial peritonitis in cirrhotic patients, severe sepsis/septic shock, and *S. aureus* bacteraemia were all associated with higher mortality. Adequate empirical treatment was increased, as well as bacteraemia of origin in the biliary and urinary tracts, which are associated with lower mortality.

**Table 4:** Evolution over periods of time of the variables associated with 30-day mortality, multivariable analyses

Characteristics	Years				
	1996–2000 OR, (95% CI)	2001–2005 OR, (95% CI)	2006–2010 OR, (95% CI)	2011–2015 OR, (95% CI)	2016–2020 OR, (95% CI)
Male	1.10, (0.85–1.43)	1.13, (0.88–1.45)	1.41, (1.12–1.79)*	1.25, (1.00–1.57)*	1.38, (1.10–1.74)*
Age	0.987, (0.98–0.99)*	0.999, (0.99–1.01)	1.007, (1.00–1.01)*	1.013, (1.01–1.02)*	1.02, (1.01–1.03)*
Severe sepsis/septic shock	0.62, (0.44–0.87)*	0.68, (0.49–0.94)*	0.76, (0.57–1.02)	0.93, (0.70–1.23)	0.70, (0.52–0.93)*
Neutropenia	0.77, (0.41–1.43)	0.80, (0.44–1.47)	0.48, (0.26–0.88)*	0.42, (0.23–0.75)*	0.36, (0.20–0.65)*
McCabe fatal-rapidly fatal	2.03, (1.52–2.70)*	1.67, (1.27–2.19)*	1.30, (1.01–1.68)*	1.68, (1.31–2.15)*	2.25, (1.75–2.89)*
Source					
Respiratory tract	1.30, (0.86–1.98)	1.26, (0.84–1.90)	1.05, (0.71–1.56)	0.85, (0.58–1.25)	0.91, (0.62–1.34)
Intra-abdominal	0.82, (0.41–1.64)	1.64, (0.90–3.01)	0.98, (0.54–1.78)	1.14, (0.65–2.01)	0.84, (0.47–1.51)
Spontaneous bacterial peritonitis in cirrhosis	1.23, (0.53–2.83)	0.63, (0.25–1.62)	0.36, (0.14–0.93)*	0.05, (0.01–0.26)*	0.06, (0.02–0.23)*
Meningitis	2.80, (1.07–7.30)*	2.27, (0.86–6.01)	1.52, (0.57–4.04)	1.06, (0.40–2.86)	1.08, (0.39–2.97)
Endocarditis	0.80, (0.49–1.31)	0.69, (0.43–1.13)	0.61, (0.39–0.95)*	0.47, (0.30–0.74)*	0.54, (0.35–0.83)*
Urinary tract	1.06, (0.72–1.55)	1.34, (0.94–1.92)	1.16, (0.85–1.60)	1.50, (1.11–2.04)*	1.67, (1.23–2.27)*
Biliary tract	1.13, (0.67–1.89)	1.43, (0.89–2.29)	1.44, (0.93–2.23)	1.93, (1.27–2.94)*	1.59, (1.04–2.43)*
Aetiology					
*S. aureus*	0.70, (0.46–1.06)	0.92, (0.62–1.36)	0.73, (0.50–1.05)	0.79, (0.55–1.13)	0.67, (0.47–0.97)*
*Enterococcus* spp.	0.96, (0.47–1.96)	0.72, (0.35–1.47)	0.58, (0.30–1.14)	0.96, (0.52–1.80)	1.01, (0.55–1.88)
*Candida* spp.	0.38, (0.12–1.20)	0.87, (0.36–2.10)	0.95, (0.42–2.15)	1.30, (0.60–2.82)	0.72, (0.32–1.61)
Polymicrobial bacteraemia	1.60, (0.93–2.68)	1.74, (1.06–2.84)*	1.93, (1.21–3.07)*	1.38, (0.87–2.17)	1.76, (1.12–2.79)*
Inmunosuppressive treatment	1.84, (1.17–2.90)*	2.53, (1.66–3.86)^*^	3.03, (2.02–4.55)*	2.65 (1,76–3.99)*	3.1, (2.09–4.6)*
Appropriate empirical antimicrobial treatment	1.20, (0.90–1.61)	1.03, (0.78–1.36)	1.13, (0.87–1.47)	1.59, (1.23–2.05)*	1.42, (1.10–1.83)*

Reference 1991–1995

*p < 0.05

## Discussion

We present the longest-running BSI cohort study and provide an updated evolution of the epidemiology of BSIs over 30 years, with significantly increasing age of patients, underlying conditions, immunosuppressive therapy, exposure to invasive procedures, and increase of CA and HA BSIs incidence. Appropriate empirical antibiotic treatment improved throughout the study period and the mortality rate decreased.

The incidence of BSI varies significantly among regions, and this in-part related to blood culture rates, population demographic differences and risk factor distribution. Our incidence is lower than that published a few years ago in Denmark, Finland and England, based on episodes of bacteraemia reported by microbiology laboratories [17, 18]. These studies reflect an increase in BSI incidence, but without adjustment for comorbidity and instrumentation as in our series. Increased age of patients often entails more comorbidities and instrumentation, a greater risk of infection with bacteraemia, and a greater probability of performing blood cultures, and this is reflected in BSIs increasing with advancing age.

The place of acquisition of BSI is akin to previous reports made in Denmark and Finland [3, 4]. Sex and age are variables that influence the comorbidities and instrumentation of patients, and therefore the source and aetiology of BSI. In this study, as in others, males were overrepresented, possibly because they are subjected to more vascular and urinary catheterisations, surgery, and immunosuppressive treatment [3, 17]. The mean age of our patients is higher than in that of a series published a few years ago [19] and increased by 11 years in patients with CA BSI and 4.5 in HA BSI. BSI due to Gram-negative microorganisms are more frequent in females and Gram-positive and polymicrobial BSIs are more frequent in males. The six most frequent pathogens (*E. coli*, *Klebsiella *spp., *S. aureus*, CoNS, *Enterococcus *spp. and *S. pneumoniae*) are in accordance with previous reports performed in other areas. However, the order and proportion of these pathogens show some differences in these studies [4, 5, 11, 20]. In CA BSI the incidence of *Salmonella tiphy* decreased, undoubtedly due to improved socio-sanitary conditions in developed countries [21]. The increase in *E. coli* stands out, as well-as an increase in urinary and biliary source of infection, and there was also an increase in *Klebsiella *spp., as in the Andalucían (Spain) series [10]. This increase in *E. coli* and *Klebsiella *spp. was associated with the increase in ESBL-producing *Enterobacteriales*. The incidence of *S. aureus* has decreased, the proportion of MRSA cases is greater than in Denmark but much lower than in the USA, and has decreased in recent years. This may be related to decreased prevalence of intravenous drug use in HIV patients and the implemented prevention and control programs [22, 23]. There were no outbreaks that may have biased the results, and carbapenemase producers were not isolated in any patients.

The proportion of HA BSI is lower than 59% in five hospitals in 2013 in Japan [24] and 53% in Ontario, Canada [9], and higher and with increasing incidence, unlike other European series [3, 4]. The most frequent cause are vascular catheters and the aetiology due to CoNS [19]. The increase in vascular catheter-associated BSIs is undoubtedly related to increased use, and possibly due to an extension in peripheral line change rules to 96 h after insertion in the last 5 years [25], or to an increase in temporary nursing staff recruitment [26]. Markedly, the appearance of the SARS-CoV-2 pandemic in the last year, was associated with an increase in vascular catheter BSI in the ICU [27].

The frequency of polymicrobial bacteraemia, its increased incidence and higher mortality than monomicrobial bacteraemia is similar to what has been already published [3, 4].

Data comparing the mortality of CA vs. HA bacteraemia is sparse. The mortality rate of our patients is within published values, and we found higher mortality in HA than in CA BSI [10, 28]. The mortality rate decreased despite the increase in age, comorbidities, immunosuppressive treatment. This is possibly due to a decrease in endocarditis and *S. aureus* bacteraemia, an increase in urinary and biliary source or appropriate empirical antibiotic treatment and improved management of haemodynamically instable patients [29, 30]. Although the mortality rate decreased, the total number of deaths increased, suggesting the possibility that greater life expectancy and comorbidities in developed countries poses a growing problem [31].

The biggest limitation of our study is that it is a single centre study, and although a multi-specialty hospital, blood culture utilization rates can differ from those of other centres or geographical areas [32]. Our results can be extrapolated given the increasing life expectancy of the population and the increase in comorbidities and instrumentation of patients is a global issue. We have not been able to quantify to what extent the improvement in survival was due to earlier detection and better management of sepsis. BSI have traditionally been classified as hospital or community-acquired, as in the Denmark, Finland, and England series. The third category of healthcare-associated BSI -widely accepted in recent years- is not assessed here [10, 11].

The greatest strength of our study is the prospective data collected for all BSI episodes by a single Infectious Diseases specialist over 30 years, having ruled out contaminating microorganisms, the high number of registered cases, adjusted for acquisition place, aetiology, comorbidities, and instrumentation of the patient that allows to see the evolution over time of the epidemiological and clinical characteristics of bacteraemia, which can help optimize management protocols for this entity.

## Conclusion

Data regarding the dynamics of invasive infections are crucial to inform the public health debate about prioritization in healthcare. The incidence of CA and HA bacteraemia increased, due to increased age, comorbidities, and instrumentation of patients, reinforcing the importance of infection control programmes and the need to strengthen infectious diseases workforce in hospitals to cope with increased complexity of patients.

## Data Availability

The datasets generated and/or analysed during the current study are not publicly available to preserve the individual privacy of the participants but they are available from the corresponding author on reasonable request.
